# Examining the complexity of functioning in persons with spinal cord injury attending first rehabilitation in Switzerland using structural equation modelling

**DOI:** 10.1038/s41393-020-0428-4

**Published:** 2020-02-13

**Authors:** Jsabel Hodel, Cristina Ehrmann, Gerold Stucki, Jerome E. Bickenbach, Birgit Prodinger, and the SwiSCI Study Group

**Affiliations:** 1 Swiss Paraplegic Research, Nottwil, Switzerland; 2 Department of Health Sciences and Medicine, University of Lucerne, Lucerne, Switzerland; 3 Faculty of Applied Health and Social Sciences, Technical University of Applied Sciences Rosenheim, Rosenheim, Germany

**Keywords:** Health care, Outcomes research

## Abstract

**Study design:**

Cross-sectional.

**Objectives:**

To examine the associations between activities, body structures and functions, and their relationship with aetiology, age and sex in persons with spinal cord injury (SCI) at discharge from first rehabilitation.

**Setting:**

Swiss SCI Cohort Study (SwiSCI).

**Methods:**

The study included 390 participants with newly acquired SCI and the International Classification of Functioning, Disability and Health (ICF) as conceptual frame of reference. Body structures were represented by injury level and severity; body functions by cardiovascular, pulmonary, skin, bowel and urinary functions and pain; mental functions by anxiety, depression, optimism and self-esteem; and activities by independence in performing activities of daily living (ADL). Using structural equation modelling (SEM), indirect effects of body structures and functions on independence in performing ADL through mental functions were tested for each mental function separately. For each structural model, fit was assessed using several indices and differences in aetiology, age and sex groups were explored.

**Results:**

The structural model about optimism showed good fit in all indices; the models about anxiety, depression and self-esteem showed conflicting fit indices, respectively. Within all models, effects on independence in performing ADL were mainly direct. Pain showed significant (*P* < 0.05) indirect effects on independence in performing ADL within the depression, optimism and self-esteem models. The model about anxiety showed differences in aetiology groups.

**Conclusions:**

Using an ICF-based modelling approach, this study presents an attempt towards a more comprehensive understanding of functioning in first rehabilitation of persons with SCI, which might be fundamental for rehabilitation planning.

## Introduction

The objective of rehabilitation is to optimise functioning for people, who because of a health condition, have difficulties carrying out activities of everyday life [1]. By ‘functioning’ we mean the key concept in the World Health Organization’s International Classification of Functioning, Disability and Health (ICF) [2], namely the sum of human body structures and functions, as well as activities and areas of participation. As the ICF makes clear, rehabilitation’s focus must be both on optimising functioning at the body level as well as the person’s capacity to perform actions and to transform this improvement in capacity by making changes in the person’s environment, to optimise their performance in everyday life. To achieve this, rehabilitation requires information on people’s functioning to guide intervention planning and, more generally, decision-making among health professionals and patients.

Spinal cord injury (SCI) is a health condition that has devastating impacts on people’s life and functioning. The injury creates impairments in body structures and functions, including the neurological damage of the spinal cord and the loss of motor, sensory and autonomic neurologic functions [3]. These impairments adversely affect the person’s independence in performing daily activities such as self-care, mobility, bladder and bowel management. Newly injured persons in acute care and first rehabilitation not only have to undergo a traumatic event, they are also placed at risk of complications such as pressure injuries, thromboembolism, cardiopulmonary arrest, cardiovascular, pulmonary and renal conditions [4]. The degree to which rehabilitation can optimise their functioning will be influenced by injury-related factors such as the cause of the injury [5], as well as sociodemographic factors [6, 7]. Factors such as depression have shown to influence functioning outcomes [8], however, how they impact the relationship between body structures and functions and activities and participation has not been examined yet. Given the wide and diverse range of impacts on body structures and functions, and resulting decrements in capacity to perform actions, SCI is associated with a high degree of complexity of people’s functioning profile.

Deepening our understanding of this complexity, and in particular the associative linkages between health condition and components of functioning will be assisting in tailoring rehabilitation so as to meet the needs of people with SCI. Moreover, as countries put regulations in place that require an ICF-based documentation of assessment (as in Switzerland where ICF-based rehabilitation goals are required for quality assurance purposes [9]), empirical investigations into the associations described by the model of the ICF are important to ensure that evidence-based decisions can be made in rehabilitation practice.

To analyse these complex association structures, statistical modelling methods can be used [10]. In the SCI literature, we have found only a few studies that use these methods and the ICF model as a framework to analyse relationship structures among components of functioning [11] and interactions with the health condition and contextual factors [12–14].

Therefore, the objective of this study is to examine the associations between activities, body structures and functions, and their relationship with contextual factors in persons with SCI. Since the Swiss SCI Cohort Study (SwiSCI) [15] was developed based on the ICF as a conceptual model, the study provides an optimal basis for our purposes. Considering the variables available in SwiSCI, the specific aims are (1) to test indirect effects of body structures and functions on activities through different mental functions, and (2) to test the resulting models for differences in aetiology, age and sex groups. We use the notion ‘indirect effects’ to account for our cross-sectional study design; it should not be used synonymously with ‘mediations’, since the latter is referring to causal hypotheses requiring longitudinal study designs [16]. In this study, body structures were specified by injury level and severity; body functions by cardiovascular, pulmonary, skin, bowel and urinary functions and pain; mental functions by anxiety, depression, optimism and self-esteem; and activities by the independence in performing activities of daily living (ADL). See Table 1 for further information.

**Table 1:** Overview of ICF concepts and corresponding operationalisations or measurement instruments and items considered within the analysis of this study.

ICF concept	Construct	Operationalization or instrument	Assessment mode	Time point	No. of items	Further specification	Response options	Item label
Body structures	Neurological level of injury	International SCI Core Data Set/ ISNCSCI [17, 45]	Clinical assessment	T4	1	Neurological level of injury	Tetraplegia (C1-C8)/paraplegia (T1-S5)^d^	Level of injury
	Severity of injury	International SCI Core Data Set/ ISNCSCI [17, 45]	Clinical assessment	T4	1	ASIA Impairment Scale	Incomplete (B, C, D)/complete (A)^d^	Severity of injury
Body functions	Bowel function	International SCI Bowel Function Basic Data Set [46]	Health record	T4	1	Normal defecation since T3	No/yes	Bowel function
	Cardiovascular function	International SCI Cardiovascular Function Basic Data Set [47]	Health record	T4	1	Occurrence of cardiovascular conditions or complications since T3	No/yes	Cardiovascular function
	Pain	International SCI Pain Basic Data Set [48]	Questionnaire ^a^ (self-reported)	T4	1	Presence of pain in the last week	No/yes	Pain
	Pulmonary function	International SCI Pulmonary Function Basic Data Set [49]	Health record	T4	1	Occurrence of pulmonary conditions or complications since T3	No/yes	Pulmonary function
	Skin function	International SCI Skin and Thermoregulation Function Basic Data Set [50]	Clinical assessment	T4	1	Presence of pressure injury since T3	No/yes	Skin function
	Urinary function	NA	Clinical assessment	T4	1	Presence of infection of urinary tract since T3	No/yes	Urinary function
	Anxiety	HADS [51, 52]	Questionnaire ^a^ (self-reported)	T4	7	Presence of anxiety symptoms in the last week	Four-level rating scales, where higher rates indicate the presence of anxiety symptoms	Stressed
								Scared
								Worried
								Relaxed
								Fearing
								Restless
								Panicked
	Depression	HADS [51, 52]	Questionnaire ^a^ (self-reported)	T4	7	Presence of depression symptoms in the last week	Four-level rating scales, where higher rates indicate the presence of depression symptoms	Enjoying as before
								Laughing
								Being cheerful
								Slowed down
								Interested in appearance
								Looking forward
								Enjoying a book
	Optimism	LOT-R [53]	Questionnaire ^a^ (self-reported)	T4	6^b^	Feeling of optimism of today	Five-level rating scales, where higher rates indicate higher optimism	Expecting the best
								Things go wrong if they can
								Optimistic about future
								Expecting things to go wrong
								Not relying on good things
								Expecting good things
	Self-esteem	RSES [54]	Questionnaire ^a^ (self-reported)	T4	4^c^	Self-esteem in general	Four-level rating scales, where higher rates indicate higher self-esteem	Having good qualities
								Feeling useless
								Being of worth
								Taking a positive attitude
Activities	Performing ADL	SCIM III [55]	Health record	T4	19	Ability of performing ADL concerning self-care, respiration, sphincter management and mobility independently	Raw sum score ranging from 0 to 100, where a higher score indicates higher independency in performing ADL	Independence in performing ADL
NA	Age at injury	NA	Health record	T1	1	Age at SCI diagnosis	Younger than or equal/older than median age in years^d^	Age
	Aetiology	International SCI Core Data Set [45]	Health record	T1	1	Cause of injury	Traumatic/non-traumatic	Aetiology
	Sex	NA	Health record	T1	1	Sex	Male/female	Sex
	Language	NA	Health record	T1	1	Language of correspondence	German/French/Italian/Other	Language

*ICF* International Classification of Functioning, Disability, and Health, *SCI* spinal cord injury, *ISNCSCI* International Standards for Neurological Classification of Spinal Cord Injury, *ASIA* American Spinal Injury Association, *T1*–*T4* Swiss SCI Inception Cohort Study measurement time points, *HADS* Hospital Anxiety and Depression Score, *LOT-R* Life Orientation Test-Revised, *RSES* Rosenberg Self-Esteem Scale, *SCIM III* Spinal Cord Independence Measure version III, *ADL* activities of daily living, *NA* not applicable.

^a^Language versions: German, French, Italian.

^b^Selection of the six non-filler items of the questionnaire.

^c^Selection of the four items of the questionnaire that were administered at more than one time point within the Swiss SCI Inception Cohort Study.

^d^Dichotomization strategy (performed after missing data imputation).

## Methods

### Study design and participants

This study used data from the SwiSCI Inception Cohort Study [15] in which newly injured persons with SCI are recruited during first rehabilitation in one of the four collaborating rehabilitation centres (SCI Center, Balgrist University Hospital, Zürich; Centre for SCI and Severe Head Injury, REHAB Basel, Basel; Clinique Romande de Réadaptation, Sion; Swiss Paraplegic Centre, Nottwil). Inclusion criteria of the SwiSCI Inception Cohort are the following: (1) age of 16 years or older, (2) permanent residence in Switzerland, (3) diagnosis of traumatic or non-traumatic SCI; exclusion criteria can be found elsewhere [15]. Measurements are performed one month (T1), three months (T2) and six months (T3) after SCI diagnosis during the clinical rehabilitation setting and at discharge (T4).

Until November 12th 2018, 883 participants were enroled in the SwiSCI Inception Cohort Study and completed data collection at discharge. For the purpose of this study, patients with the following characteristics were excluded from the sample in specific order: (1) death during first rehabilitation (*N* = 16), (2) no observations in all items of the independence in performing ADL measure at T4 (*N* = 174), (3) no observations in all items of the measures of the mental functions at T4 (*N* = 290), (4) intact neurological level or normal degree of impairment [17] at T4 (*N* = 13).

### Measures

The SwiSCI builds upon the ICF as conceptual foundation and during its development, instruments to operationalise the components of the ICF were identified [18, 19]. The ICF concepts reflected within the present study, measurement information, corresponding variables and response options are shown in Table 1.

### Missing data imputation

Observations in the response options ‘unknown’ or ‘unable to determine’ were considered as missing. Missing observations of the injury level or severity at T4 were replaced by the last observation of the corresponding variable at T3 or T2 or T1. Missing observations in the other variables were replaced by using the non-parametric random forest method MissForest [20] which is able to handle data with continuous as well as categorical variables. The MissForest method has been shown to not only outperform established methods such as nearest neighbour imputation and multivariate imputation by using chained equations [20, 21], but also other random forest imputation methods [22]. See Supplementary Table 1 for further information on missing observations before data imputation.

### Rasch measurement model for the independence in performing ADL

Using the Rasch measurement model [23, 24], the raw sum score of the Spinal Cord Independence Measure version III (SCIM III) was transformed to an interval sum score. Model fit was assessed by the individual and overall item fit, the person fit and the *P* value of the *χ*^2^ test statistic of the item–trait interaction with good fit for non-significant *χ*^2^ (*P* > 0.05). Score reliability was tested by the person separation index (PSI) with an adequate expectation of 0.70 or above at the group level. To test whether the data fulfils the underlying model assumptions, local independency among items, unidimensionality of the score and the absence of differential item functioning (DIF) were tested iteratively. If items showed local dependence, a testlet approach was used to introduce super-items created by summing the initial response options of local dependent items. The corresponding analysis approach is described elsewhere [25].

### Measurement models for the mental functions

We hypothesised each mental function to be a single latent factor represented by the respective observed questionnaire items (indicators) with uncorrelated measurement errors. In this context, direct effects of latent factors on indicators are referred to as factor loadings. Confirmatory factor analysis (CFA) [16, 26] was used to test if the hypothesised measurement models fit the data and hence, represent a single latent factor. Model fit was assessed by the following fit indices: *χ*^2^ test statistic, comparative fit index (CFI), root-mean-square error of approximation (RMSEA) and weighted-root-mean-square residuals (WRMR). The criteria to evaluate goodness of model fit were: non-significant *χ*^2^ (*P* > 0.05), CFI > 0.95, RMSEA < 0.05 and WRMR < 1.0 [27]. If the initial CFA did not show good fit, the modification indices (MI) and residual correlation matrix of the respective measurement model were examined and indicator error correlations were introduced iteratively, (1) starting from the largest MI with significant Bonferroni-adjusted *P* value, and (2) starting from the largest absolute residual correlation >0.10 [16]. In the final measurement models, only significant indicator error correlations were retained.

For all measurement models, invariance was tested on the level of the significance pattern (configural invariance) and the estimates (weak invariance) of the factor loadings for aetiology, age, sex, level and severity of injury and language (German, French) groups as described by Hirschfeld and von Brachel [28].

### Structural models

By using structural equation modelling (SEM) [16], indirect effects of body structures and functions on the independence in performing ADL through the mental functions anxiety, depression, optimism and self-esteem were tested for each mental function separately. Starting from the biopsychosocial model underlying the ICF, the following considerations guided the development of these hypotheses: first, we assumed the effects of body structures and functions on activities to be the primary or focal relationship within first rehabilitation of persons with SCI, and this relationship and patient’s state of health to be most stable at the point of discharge. Therefore, we have applied data from discharge. Second, we considered anxiety, depression, optimism and self-esteem as mental functions belonging to the ICF component of body structures and functions. Since body structures and functions can be influenced by other body structures and functions, we hypothesised possible indirect effects of the other body structures and functions on activities through the mental functions. Third, any variables on environmental factors were not considered in this study since we draw upon data collected in first rehabilitation settings which we assumed to be not significantly different in their setup. Any differences would be a reflection of differences related to the rehabilitation setting rather than the person’s environment. Fourth, any variables on participation in life of persons with SCI were not considered in this study since we assumed that a meaningful participation indicator requires a follow-up time after first rehabilitation.

For the SEM, the interval sum score of the SCIM III and the measurement models for the mental functions as resulted from the previous analyses were used. Model fit was assessed by the *χ*^2^ test statistic, the CFI and the WRMR. The following criteria were used to evaluate goodness of model fit: non-significant *χ*^2^ (*P* > 0.05), CFI > 0.95 and WRMR < 0.90 [27].

Each structural model was explored for differences in aetiology, age and sex groups, provided that the measurement model for the corresponding mental function showed invariance for the respective group variable [29]. Whether a structural model shows differences in a specific group variable was assessed by comparing the *χ*^2^ test statistics between the corresponding freed structural model (allowing path parameters of the model to differ across respective groups) and the corresponding constrained structural model (restricting path parameters to be the same across respective groups).

The Rasch analyses were performed using *RUMM2030* [30], other analyses were conducted by using *R 3.5.0* [31]. Imputation of missing observations was undertaken by the use of the package *missForest 1.4* [20]. CFA and SEM were conducted by using the package *lavaan 0.6–3* [32] and its weighted least squares mean- and variance-adjusted estimator able to compute robust standard errors of the model parameters and mean- and variance-adjusted test statistics. If not explicitly stated other, the significance level of *P* values refers to 0.05.

## Results

In total, 390 participants were considered within this study. Sample descriptive information are presented in Table 2. Participants were mainly male (69.49%) with incomplete (83.59% after missing data imputation) paraplegia (60.77% after missing data imputation). Mean age was 53.82 years (s.d. = 16.47) and median length of stay in first rehabilitation was 133.5 days (25–75% percentiles = 75.25–192.5 days). The observed variance–covariance matrix among the imputed model relevant variables is presented in Supplementary Table 2.

**Table 2:** Characteristics of SwiSCI Inception Cohort Study participants and participants included within this study.

Characteristics	SwiSCI Inception Cohort Study (*N* = 883)	Present study before missing data imputation (*N* = 390)	*P* value
Sex			0.43
Female (%)	289 (32.73)	119 (30.51)	
Male (%)	594 (67.27)	271 (69.49)	
Missing (%)	0 (0)	0 (0)	
Mean age at SCI diagnosis, years (s.d.)	55.57 (18.44)	53.82 (16.47)	<0.05
Median age at SCI diagnosis, years (1./3. quantiles)	58 (43/71)	55 (42/67)	
Younger than or equal median age (%)	435 (49.26)	196 (50.26)	
Older than median age (%)	448 (50.74)	194 (49.74)	
Missing (%)	0 (0)	0 (0)	
Median length of stay, days (1./3. quantiles)	126 (67/185.5)	133.5 (75.25/192.5)	0.06
Missing (%)	0 (0)	0 (0)	
Language of correspondence			0.99
German (%)	678 (76.78)	299 (76.67)	
French (%)	172 (19.48)	78 (20.00)	
Italian (%)	23 (2.60)	11 (2.82)	
Other (%)	3 (0.34)	2 (0.51)	
Missing (%)	7 (0.79)	0 (0)	
Aetiology			0.47
Traumatic (%)	497 (56.29)	228 (58.46)	
Non-traumatic (%)	386 (43.71)	162 (41.54)	
Missing (%)	0 (0)	0 (0)	
Level of injury at discharge			<0.001
Tetraplegia (%)	271 (30.69)	152 (38.97)	
Paraplegia (%)	436 (49.38)	235 (60.26)	
Intact (%)	28 (3.17)	0 (0)	
Missing (%)	148 (16.76)	3 (0.77)	
Severity of injury at discharge			0.22
Complete (%)	140 (15.86)	63 (16.15)	
Incomplete (%)	586 (66.36)	324 (83.08)	
Missing (%)	157 (17.78)	3 (0.77)	
AIS-based neurological groups at discharge			<0.05
C1–4 AIS A, B or C (%)	41(4.64)	16 (4.10)	
C5–8 AIS A, B or C (%)	39 (4.42)	19 (4.87)	
T1-S5 AIS A, B or C (%)	162 (18.35)	80 (20.51)	
AIS D (%)	455 (51.53)	271 (69.49)	
AIS E (%)	27 (3.06)	0 (0)	
Missing (%)	159 (18.01)	4 (1.03)	

Distribution equality tests were performed using Pearson’s *χ*^2^ test (without continuity correction) for categorical variables and Mann–Whitney test (without continuity correction) for continuous variables.

*AIS* American Spinal Injury Association Impairment Scale, *SCI* spinal cord injury, *SwiSCI* Swiss Spinal Cord Injury Cohort Study.

### Rasch measurement model for the independence in performing ADL

For the final model, two testlets were created: one testlet incorporated the items of the self-care subscale and the respiration and sphincter management subscale, the other testlet incorporated the items of the mobility subscale of the SCIM III. This testlet design showed good model fit with *χ*^2^ = 18.28 (df = 10, *P* = 0.05) and PSI (with extremes) = 0.92. Moreover, no DIF has been present for aetiology, age and sex.

### Measurement models for the mental functions

None of the measurement models for the mental functions showed good fit in all indices in the initial CFA. The final model fit statistics after introducing indicator error correlations are reported in the following paragraph.

Anxiety: no indicator error correlations were introduced according to the pre-defined criteria. The initial CFA model was retained and showed good model fit in two of four indices with *χ*^2^ = 33.328 (df = 14, *P* = 0.003), CFI = 0.992, RMSEA = 0.060, WRMR = 0.588; depression: after introducing one indicator error correlation, the model showed good fit in two of four indices with *χ*^2^ = 40.112 (df = 13, *P* = 0.000), CFI = 0.990, RMSEA = 0.073, WRMR = 0.661; optimism: after introducing four indicator error correlations, the final model showed good fit in all indices with *χ*^2^ = 9.056 (df = 5, *P* = 0.107), CFI = 0.998, RMSEA = 0.046, WRMR = 0.285; self-esteem: after introducing one indicator error correlation, the model showed good fit in two of four indices with *χ*^2^ = 6.961 (df = 1, *P* = 0.008), CFI = 0.994, RMSEA = 0.124, WRMR = 0.417.

The final measurement models for the mental functions including estimated factor loadings and indicator error correlations are shown in Supplementary Fig. 1. Model parameter estimates are presented completely standardised. Thus, the interpretation of the factor loadings is the following: given a change by one standard deviation unit in the latent factor, each factor loading estimates the corresponding amount of change in standard deviation units in the latent response variable assumed to underlie the respective observed indicator [16]. The factor loadings furthermore estimate the Pearson correlation between latent factor and respective latent response variable and their squares indicate the proportion of explained variance (*R*^2^) of the latent factor by the latent response variables [16].

The residual correlation matrices indicating the difference between observed and model-implied correlations for each final model are shown in the Supplementary Table 3.

The full results of the invariance tests of the measurement models can be found in Supplementary Table 4. Within this section we only present the results relevant for the subsequent group difference tests of the structural models. At the level of factor loading estimates, the anxiety and depression measurement models are both invariant for aetiology, age and sex groups, the optimism measurement model is invariant for age groups, and the self-esteem measurement model is invariant for age and sex groups.

### Structural models

The model fit statistics of the hypothesised structural models are reported in the following paragraph.

Anxiety: the model showed good fit in two of three indices with *χ*^2^ = 120.030 (df = 82, *P* = 0.004), CFI = 0.983, WRMR = 0.811; depression: the model showed good fit in two of three indices with *χ*^2^ = 107.704 (df = 81, *P* = 0.025), CFI = 0.990, WRMR = 0.770; optimism: the model showed overall good fit with *χ*^2^ = 60.360 (df = 62, *P* = 0.535), CFI = 1.000, WRMR = 0.588; self-esteem: the model showed good fit in two of three indices with *χ*^2^ = 67.077 (df = 36, *P* = 0.001), CFI = 0.971, WRMR = 0.825. The residual correlation matrices for each model are shown in the Supplementary Table 5.

**Fig. 1:**
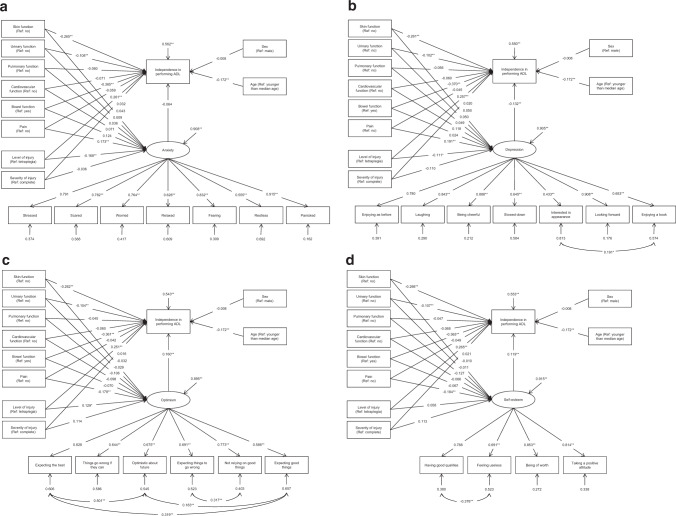
Structural models showing the relationships of body structures and functions with activities of daily living. **a** Completely standardised parameter estimates of the structural model about anxiety (*N* = 390). **b** Completely standardised parameter estimates of the structural model about depression (*N* = 390). **c** Completely standardised parameter estimates of the structural model about optimism (*N* = 390). **d** Completely standardised parameter estimates of the structural model about self-esteem (*N* = 390). *ADL* activities of daily living, *Ref* reference response option of binary variables. Squares indicate observable variables including the independence in performing activities of daily living (ADL) specified by the interval sum score of the Spinal Cord Independence Measure version III (SCIM III); ellipses indicate latent factors; single-headed arrows indicate direct effects including measurement errors; double-headed arrows indicate correlations; correlations among and measurement errors of independent observable variables are omitted; *P < 0.05; **P < 0.01.

The structural models and completely standardised parameter estimates are shown in Fig. 1a–d. The interpretation of factor loadings is the same as already described in the previous section; the interpretation of the other path coefficients is analogous to the interpretation of coefficients in a multiple regression: given a change of one standard deviation unit in the independent variable, the path coefficient estimates the corresponding change in standard deviation units in the dependent variable, holding all other respective independent variables constant. Fig. 1c for example indicates that the presence of a pressure injury (skin function, response option yes) is associated with lower independence in performing ADL (path coefficient *β* = −0.262, *P* < 0.01) and lower optimism (*β* = −0.032); whereas a lower level of injury (paraplegia) is associated with higher independence in performing ADL (*β* = 0.251, *P* < 0.01) and higher optimism (*β* = 0.129, *P* < 0.05); and higher optimism is associated with higher independence in performing ADL (*β* = 0.160, *P* < 0.01). When looking at the squared factor loadings in this model, we see that the latent response variables represented by the indicator variables show proportions of explained variance of the latent factor optimism between 0.34 (‘expecting good things’) and 0.60 (‘not relying on good things’).

The respective model estimates for the indirect and total (direct plus indirect) effects of body structures and functions on the independence in performing ADL for the four structural models are shown in Table 3. Within all structural models, effects on independence in performing ADL were mainly direct with significant positive effects of a lower level of injury and significant negative effects of occurring complications or conditions in urinary, bowel and skin functions. Significant indirect effects were found for pain within the structural models about depression, optimism and self-esteem, respectively.

**Table 3:** Completely standardised estimates for the indirect and total effects of body structures and functions on the independence in performing activities of daily living for the structural models about anxiety, depression, optimism and self-esteem.

ICF concept and variable	Indirect effects through:				Total effects
	Anxiety	Depression	Optimism	Self-esteem	
Body functions					
Bowel function (Ref: yes)	−0.008	−0.003	−0.011	−0.008	−0.373**
Cardiovascular function (Ref: no)	−0.005	−0.016	−0.016	−0.008	−0.076
Pain (Ref: no)	−0.011	−0.025*	−0.029*	−0.022*	−0.070
Pulmonary function (Ref: no)	−0.002	−0.006	−0.017	−0.015	−0.062
Skin function (Ref: no)	−0.003	−0.007	−0.005	−0.001	−0.267**
Urinary function (Ref: no)	−0.001	−0.007	−0.005	−0.001	−0.109**
Body structures					
Level of injury (Ref: tetraplegia)	0.011	0.015	0.021	0.007	0.272**
Severity of injury (Ref: complete)	0.002	0.015	0.018	0.013	0.035

The total effects are the same for all structural models.

*Ref* reference response option of binary variables.

*P  <  0.05; **P  <  0.01.

Table 4 shows the results of the structural model group difference tests. Significant group differences were found in aetiology groups for the structural model about anxiety.

**Table 4:** Group difference tests of the structural models for aetiology, age and sex groups.

Structural model and parameter constrains	${{\upchi}}_{\mathrm{M}}^{2}$	${\mathrm{df}}_{\mathrm{M}}$	Model comparison	
			${\upchi}_{\mathrm{D}}^2$	${\mathrm{df}}_{\mathrm{D}}$
**Aetiology**				
Anxiety				
Freed	134.939	164		
Constrained	207.347	183	31.157*	19
Depression				
Freed	130.289	162		
Constrained	172.903	181	20.509	19
Optimism				
Freed	–	–		
Constrained	–	–	–	–
Self-esteem				
Freed	–	–		
Constrained	–	–	–	–
**Age**				
Anxiety				
Freed	133.634	150		
Constrained	188.145	168	24.617	18
Depression				
Freed	113.641	148		
Constrained	140.591	166	13.927	18
Optimism				
Freed	72.815	112		
Constrained	112.368	130	20.790	18
Self-esteem				
Freed	64.630	64		
Constrained	89.555	82	15.876	18
**Sex**				
Anxiety				
Freed	96.295	150		
Constrained	127.982	168	16.770	18
Depression				
Freed	100.820	148		
Constrained	126.733	166	15.230	18
Optimism				
Freed	–	–		
Constrained	–	–	–	–
Self-esteem				
Freed	53.255	64		
Constrained	107.167	82	27.832	18

Freed, no constrains on parameter estimates across respective groups; Constrained, all path estimates constrained to be equal across respective groups. *M* model, *D* difference.

^*^P  <  0.05.

## Discussion

Using SEM to examine the possible influence of mental functions within the relationship of body structures, body functions and activities, pain showed significant indirect effects on the independence in performing activities of ADL in the structural models about depression, optimism and self-esteem. Group differences were found in aetiology groups for the structural model about anxiety.

However, the results need to be interpreted within its conceptual framework and the cross-sectional design of the study: first, personal factors are not classified yet in the ICF and there remains to be a debate about their definition and relationship to mental functions [33]. Regardless whether you consider anxiety, depression, optimism and self-esteem as mental functions or personal factors, they are important when looking at peoples’ functioning. Second, this study reflects an attempt towards generating empirical evidence for a comprehensive understanding of functioning in first rehabilitation of persons with SCI as it is shown in the ICF. In this understanding, it can serve as a starting point for further model development and analyses. Since pain is the only body function that showed indirect effects on independence in performing ADL in the structural models about depression, optimism and self-esteem, it could be worthwhile to reconsider the relationship of pain and these mental functions in more detail and together with other pain items, e.g. clinical pain records.

The community survey of SwiSCI revealed that pain is highly prevalent in persons with SCI living in the community (with musculoskeletal type of pain most frequently reported) [34] and is perceived as one of the most important problems in functioning following SCI [35]. However, the relationships among pain, mental functions and independence in performing ADL appear to be complex, as for example literature about the pain–depression relationship often reflects both directions: in the general population, pain and depression symptoms are found to be commonly occurring and their relationship seem to be bidirectional [36]. Moreover, the bidirectional associations between depressive symptoms and pain seem to be similar for people with functioning problems and those without [37]. In the SCI community setting, increased pain was found to be a risk factor for developing of depression [38]. Moreover, chronic pain is suggested to be associated with increased depressive symptom levels and less participation [39], and with negative effects on psychological functioning, social integration and activities including mobility, self-care, social and recreational activities [40]. On the other hand, a meta-analysis of possible determinants for pain in persons with SCI has shown that depression prevalence is associated with pain prevalence [41]. Within the acute SCI setting, the pain–depression interaction remains unclear; different models have been tested and are conceivable [42], other studies have found that depressive symptoms are not related to pain or functional impairment [43]. Therefore, further research is needed to uncover comprehensive interactions among mental functions, possible changes in mental functions over time, and their associations with other body functions, body structures, activities and participation [44].

### Limitations

We note several methodological limitations to our study. First, the three measurement models for anxiety, depression and self-esteem are lacking good fit in terms of the *P* value of the *χ*^2^ test statistic which is leading to unknown bias in the corresponding structural models, which likewise are lacking good fit in this index. Second, since the measurement models for the mental functions were modified in an exploratory and data-driven way by introducing indicator error correlations based on MI and residual correlation matrices, the results of this study are not generalisable and should be cross-validated. Moreover, indicator error correlations can be viewed as shared variance besides the common latent factor and the measurement models become multidimensional by their introduction. Third, we might not be able to detect invariances within our measurement models or group differences within our structural models due to the small sample sizes of some groups tested. Fourth, a selection bias on the sample used in this analysis could have occurred since (1) the filling in of the questionnaires within the SwiSCI Inception Cohort Study is optional and (2) we excluded participants with no observations in the ADL and the mental functions variables. Fifth, the cross-sectional design of the study does not allow for causal conclusions. Thus, a longitudinal study design is needed to clarify and extend the presented structural models.

## Conclusion

Using an ICF-based modelling approach, this study presents an attempt towards a more comprehensive understanding of functioning in first rehabilitation of persons with SCI, which might be fundamental for rehabilitation planning and decision-making among health professionals and patients.

## Supplementary information

Supplementary Information

## Data Availability

The datasets generated and analysed during this study are not publicly available due to the commitment of SwiSCI to protect participants’ privacy but are available at the SwiSCI Study Center (swisci.research@paraplegie.ch) on reasonable request.
