# Correction: Examining the complexity of functioning in persons with spinal cord injury attending first rehabilitation in Switzerland using structural equation modelling

**DOI:** 10.1038/s41393-020-0464-0

**Published:** 2020-04-08

**Authors:** Jsabel Hodel, Cristina Ehrmann, Gerold Stucki, Jerome E. Bickenbach, Birgit Prodinger, and the SwiSCI Study Group

**Affiliations:** 1 Swiss Paraplegic Research, Nottwil, Switzerland; 2 Department of Health Sciences and Medicine, University of Lucerne, Lucerne, Switzerland; 3 Faculty of Applied Health and Social Sciences, Technical University of Applied Sciences Rosenheim, Rosenheim, Germany

**Keywords:** Health care, Outcomes research

Correction to: *Spinal Cord*

10.1038/s41393-020-0428-4 published online 13 February 2020

Following publication of this article, the authors noticed an error due to incorrect naming of two groups: ‘paraplegia’ and ‘tetraplegia’. This affects Tables 1 and 2, and the Results section. The correction is as following: In Table 1 ‘**Paraplegia** (C1-C8)/**tetraplegia** (T1-S5)’ has now been corrected to ‘**Tetraplegia** (C1-C8)/**paraplegia** (T1-S5)’. In the first paragraph of the Results section ‘Participants were mainly male (69.49%) with incomplete (83.59% after missing data imputation) **tetraplegia** (60.77% after missing data imputation)’, has now been changed to ‘Participants were mainly male (69.49%) with incomplete (83.59% after missing data imputation) **paraplegia** (60.77% after missing data imputation)’. Finally, in Table 2, ‘Paraplegia’ and ‘Tetraplegia’ have been swapped to align with the correct, corresponding values. This has been corrected in both the PDF and HTML versions of the article, and does not change the interpretation of the data.

