# Supplementary material for: Examining the complexity of functioning in persons with spinal cord injury attending first rehabilitation in Switzerland using structural equation modelling
Source: Spinal Cord. 2020 Feb 13;58(5):570–80. doi: 10.1038/s41393-020-0428-4 (PMC7214256; doi:10.1038/s41393-020-0428-4)
Supplement: Supplementary file 1 — Supplementary Information [file 41393_2020_428_MOESM1_ESM.pdf]

Legend of supplementary material

Supplementary Table 1: Analysis relevant variables and number of missing observations before data imputation

Supplementary Table 2: Observed analysis relevant variance-covariance matrix (N=390)

Supplementary Figure 1: Completely standardized parameter estimates of the measurement models for a) anxiety, b) depression, c) optimism and d) self-esteem (N=390)

Supplementary Table 3: Residual correlation matrices of the final measurement models for the mental functions anxiety, depression, optimism and self-esteem (N=390)

Supplementary Table 4: Measurement invariance tests of the measurement models for aetiology, age, sex, level and severity of injury and language (German, French) groups

Supplementary Table 5: Residual correlation matrices of the structural models (N=390)

Supplementary Table 1 Analysis relevant variables and number of missing observations before data imputation

Variable	Present study before imputation (N=390)
<u>Body structures</u>	
Level of injury at discharge	
Paraplegia (%)	152 (38.97)
Tetraplegia (%)	235 (60.26)
Missing (%)	3 (0.77)
Severity of injury at discharge	
Complete (%)	63 (16.15)
Incomplete (%)	324 (83.08)
Missing (%)	3 (0.77)
<u>Body functions</u>	
Bowel function	
No (%)	189 (48.46)
Yes (%)	181 (46.41)
Missing (%)	20 (5.13)
Cardiovascular function	
No (%)	303 (77.69)
Yes (%)	85 (21.79)
Missing (%)	2 (0.51)
Pain	
No (%)	135 (34.62)
Yes (%)	244 (62.56)
Missing (%)	11 (2.82)
Pulmonary function	
No (%)	346 (88.72)
Yes (%)	41 (10.51)
Missing (%)	3 (0.77)
Skin function	
No (%)	343 (87.95)
Yes (%)	42 (10.77)
Missing (%)	5 (1.28)
Urinary function	
No (%)	286 (73.33)
Yes (%)	103 (26.41)
Missing (%)	1 (0.26)
<u>Mental functions</u>	
Anxiety: Stressed	
0 (%)	133 (34.10)
1 (%)	188 (48.21)
2 (%)	47 (12.05)
3 (%)	14 (3.59)
Missing (%)	8 (2.05)
Anxiety: Scared	
0 (%)	207 (53.08)
1 (%)	103 (26.41)
2 (%)	51 (13.08)
3 (%)	20 (5.13)
Missing (%)	9 (2.31)

Abbreviations: ADL, activities of daily living; AIS, American Spinal Injury Association Impairment Scale; SCI, spinal cord injury; SwiSCI, Swiss Spinal Cord Injury Cohort Study; NA, not applicable.

Supplementary Table 1 Continued

Variable	Present study before imputation (N=390)
Anxiety: Worried	
0 (%)	174 (44.62)
1 (%)	140 (35.90)
2 (%)	53 (13.59)
3 (%)	15 (3.85)
Missing (%)	8 (2.05)
Anxiety: Relaxed	
0 (%)	142 (36.41)
1 (%)	166 (42.56)
2 (%)	65 (16.67)
3 (%)	9 (2.31)
Missing (%)	8 (2.05)
Anxiety: Fearing	
0 (%)	198 (50.77)
1 (%)	154 (39.49)
2 (%)	21 (5.38)
3 (%)	8 (2.05)
Missing (%)	9 (2.31)
Anxiety: Restless	
0 (%)	159 (40.77)
1 (%)	133 (34.10)
2 (%)	76 (19.49)
3 (%)	14 (3.59)
Missing (%)	8 (2.05)
Anxiety: Panicked	
0 (%)	267 (68.46)
1 (%)	93 (23.85)
2 (%)	14 (3.59)
3 (%)	6 (1.54)
Missing (%)	10 (2.56)
Depression: Enjoying as before	
0 (%)	127 (32.56)
1 (%)	176 (45.13)
2 (%)	61 (15.64)
3 (%)	14 (3.59)
Missing (%)	12 (3.08)
Depression: Laughing	
0 (%)	225 (57.69)
1 (%)	121 (31.03)
2 (%)	30 (7.69)
3 (%)	6 (1.54)
Missing (%)	8 (2.05)
Depression: Being cheerful	
0 (%)	205 (52.56)
1 (%)	128 (32.82)
2 (%)	37 (9.49)
3 (%)	11 (2.82)
Missing (%)	9 (2.31)

Abbreviations: ADL, activities of daily living; AIS, American Spinal Injury Association Impairment Scale; SCI, spinal cord injury; SwiSCI, Swiss Spinal Cord Injury Cohort Study; NA, not applicable.

Supplementary Table 1 Continued

Variable	Present study before imputation (N=390)
Depression: Slowed down	
0 (%)	74 (18.97)
1 (%)	200 (51.28)
2 (%)	65 (16.67)
3 (%)	42 (10.77)
Missing (%)	9 (2.31)
Depression: Interested in appearance	
0 (%)	264 (67.69)
1 (%)	68 (17.44)
2 (%)	38 (9.74)
3 (%)	11 (2.82)
Missing (%)	9 (2.31)
Depression: Looking forward	
0 (%)	181 (46.41)
1 (%)	134 (34.36)
2 (%)	51 (13.08)
3 (%)	15 (3.85)
Missing (%)	9 (2.31)
Depression: Enjoying a book	
0 (%)	250 (64.10)
1 (%)	90 (23.08)
2 (%)	19 (4.87)
3 (%)	19 (4.87)
Missing (%)	12 (3.08)
Optimism: Expecting the best	
0 (%)	4 (1.03)
1 (%)	21 (5.38)
2 (%)	69 (17.69)
3 (%)	116 (29.74)
4 (%)	159 (40.77)
Missing (%)	21 (5.38)
Optimism: Things go wrong if they can	
0 (%)	22 (5.64)
1 (%)	54 (13.85)
2 (%)	79 (20.26)
3 (%)	113 (28.97)
4 (%)	97 (24.87)
Missing (%)	25 (6.41)
Optimism: Optimistic about future	
0 (%)	8 (2.05)
1 (%)	22 (5.64)
2 (%)	68 (17.44)
3 (%)	124 (31.79)
4 (%)	147 (37.69)
Missing (%)	21 (5.38)
Optimism: Expecting things to go wrong	
0 (%)	20 (5.13)
1 (%)	56 (14.36)
2 (%)	61 (15.64)
3 (%)	127 (32.56)
4 (%)	104 (26.67)
Missing (%)	22 (5.64)

Abbreviations: ADL, activities of daily living; AIS, American Spinal Injury Association Impairment Scale; SCI, spinal cord injury; SwiSCI, Swiss Spinal Cord Injury Cohort Study; NA, not applicable.

Supplementary Table 1 Continued

Variable	Present study before imputation (N=390)
Optimism: Not relying on good things	
0 (%)	14 (3.59)
1 (%)	50 (12.82)
2 (%)	57 (14.62)
3 (%)	114 (29.23)
4 (%)	134 (34.36)
Missing (%)	21 (5.38)
Optimism: Expecting good things	
0 (%)	16 (4.10)
1 (%)	34 (8.72)
2 (%)	70 (17.95)
3 (%)	114 (29.23)
4 (%)	128 (32.82)
Missing (%)	28 (7.18)
Self-esteem: Having good qualities	
0 (%)	0 (0)
1 (%)	8 (2.05)
2 (%)	207 (53.08)
3 (%)	166 (42.56)
Missing (%)	9 (2.31)
Self-esteem: Feeling useless	
0 (%)	17 (4.36)
1 (%)	61 (15.64)
2 (%)	81 (20.77)
3 (%)	218 (55.90)
Missing (%)	13 (3.33)
Self-esteem: Being of worth	
0 (%)	9 (2.31)
1 (%)	20 (5.13)
2 (%)	137 (35.13)
3 (%)	211 (54.10)
Missing (%)	13 (3.33)
Self-esteem: Taking a positive attitude	
0 (%)	9 (2.31)
1 (%)	23 (5.90)
2 (%)	156 (40.00)
3 (%)	193 (49.49)
Missing (%)	9 (2.31)
Activities	
Mean independence in performing ADL (s.d.)	89.46 (11.28)
Missing (%)	0 (0)
Others	
Mean age at SCI diagnosis, years (s.d.)	53.82 (16.47)
Missing (%)	0 (0)
Aetiology	
Traumatic (%)	228 (58.46)
Non-traumatic (%)	162 (41.54)
Missing (%)	0 (0)

Abbreviations: ADL, activities of daily living; AIS, American Spinal Injury Association Impairment Scale; SCI, spinal cord injury; SwiSCI, Swiss Spinal Cord Injury Cohort Study; NA, not applicable.

Supplementary Table 1 Continued

Variable	Present study before imputation (N=390)
Sex	
Female (%)	119 (30.51)
Male (%)	271 (69.49)
Missing (%)	0 (0)
Language of correspondence	
German (%)	299 (76.67)
French (%)	78 (20.00)
Italian (%)	11 (2.82)
Other (%)	2 (0.51)
Missing (%)	0 (0)

Abbreviations: ADL, activities of daily living; AIS, American Spinal Injury Association Impairment Scale; SCI, spinal cord injury; SwiSCI, Swiss Spinal Cord Injury Cohort Study; NA, not applicable.

Supplementary Table 2 Observed analysis relevant variance-covariance matrix (N=390)

Variable	1.	2.	3.	4.	5.	6.	7.	8.	9.	10.	11.	12.	13.	14.	15.	16.	17.	18.	19.	20.	21.	22.	23.	24.	25.	26.	27.	28.	29.	30.	31.	32.	33.	34.	35.
1. Age at SCI diagnosis	0.25																																		
2. Sex	0.00	0.21																																	
3. Level of injury	-0.02	0.02	0.24																																
4. Severity of injury	0.03	0.02	-0.02	0.14																															
5. Bowl function	-0.01	-0.02	0.03	-0.08	0.25																														
6. Cardiovascular function	0.05	0.00	0.02	0.02	-0.02	0.17																													
7. Pain	0.01	0.01	-0.03	0.02	0.00	0.02	0.23																												
8. Pulmonary function	0.02	0.00	-0.01	0.01	0.00	0.03	0.01	0.09																											
9. Skin function	0.02	0.00	0.00	-0.03	0.03	0.02	0.00	0.01	0.10																										
10. Urinary function	-0.01	-0.01	0.01	-0.03	0.06	0.01	-0.01	0.00	0.03	0.19																									
11. Stressed											1.00																								
12. Scared											0.55	1.00																							
13. Worried											0.64	0.57	1.00																						
14. Relaxed											0.53	0.44	0.43	1.00																					
15. Fearing											0.61	0.69	0.62	0.53	1.00																				
16. Restless											0.46	0.39	0.39	0.41	0.36	1.00																			
17. Panicked											0.71	0.70	0.68	0.57	0.77	0.46	1.00																		
18. Enjoying as before																		1.00																	
19. Laughing																		0.68	1.00																
20. Being cheerful																		0.65	0.75	1.00															
21. Slowed down																		0.46	0.43	0.53	1.00														
22. Interested in appearance																		0.42	0.33	0.36	0.22	1.00													
23. Looking forward																		0.73	0.72	0.79	0.62	0.38	1.00												
24. Enjoying a book																		0.39	0.54	0.59	0.43	0.39	0.56	1.00											
25. Expecting the best																									1.00										
26. Things go wrong																									0.39	1.00									
27. Optimistic about future																									0.71	0.40	1.00								
28. Expecting things to go wrong																									0.38	0.45	0.50	1.00							
29. Not relying on good things																									0.50	0.48	0.49	0.66	1.00						
30. Expecting good things																									0.57	0.36	0.50	0.38	0.45	1.00					
31. Having good qualities																															1.00				
32. Feeling useless																															0.36	1.00			
33. Being of worth																															0.69	0.51	1.00		
34. Taking positive attitude																															0.59	0.56	0.66	1.00	
35. Independence in performing activities of daily living											-1.59	-1.78	-1.56	-1.79	-1.42	0.17	-2.23	-2.69	-2.57	-2.34	-1.30	-1.70	-2.50	-0.99	2.48	1.95	2.48	2.19	1.91	1.32	1.03	3.47	1.92	1.24	127.01

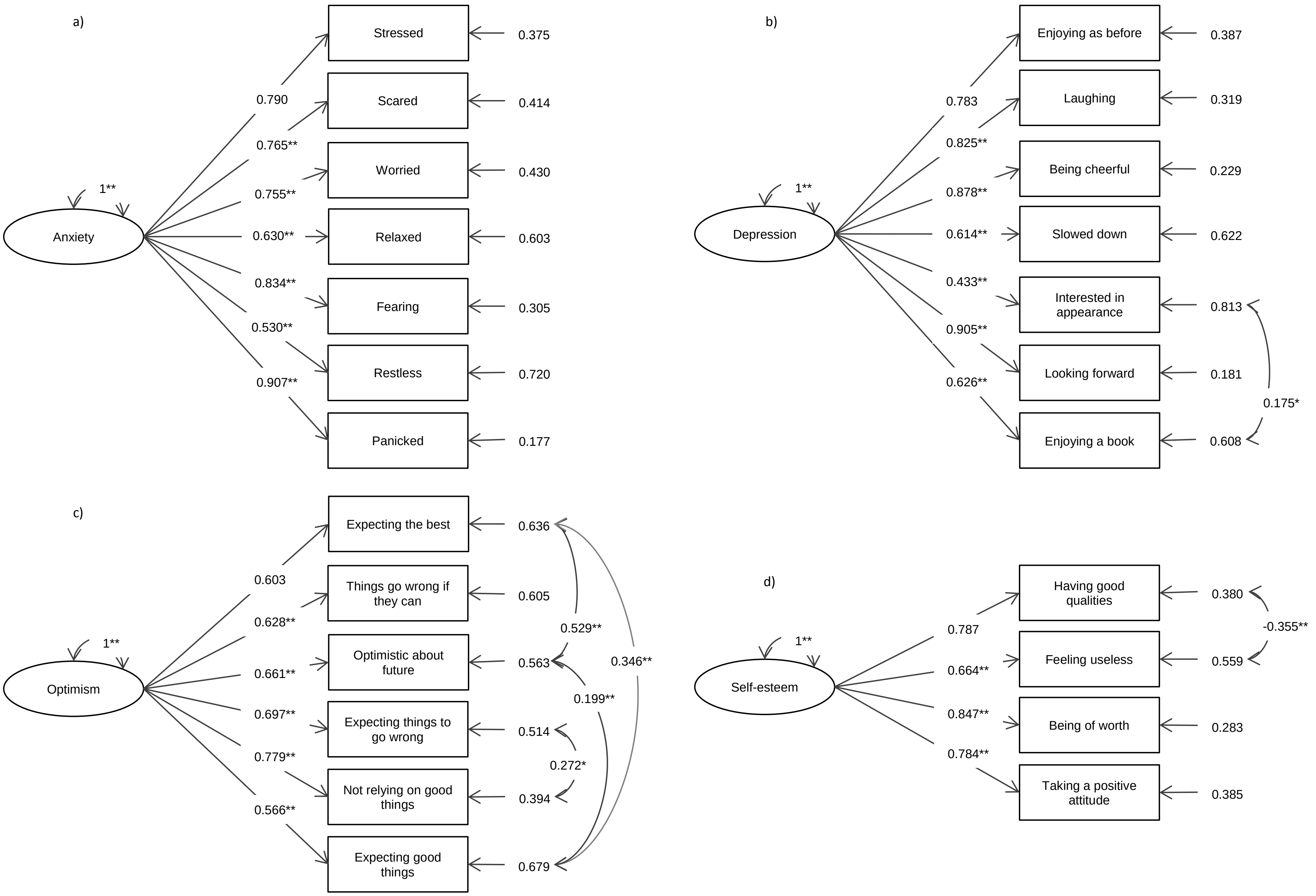

Supplementary Figure 1 Completely standardized parameter estimates of the measurement models for (a) anxiety, (b) depression, (c) optimism and (d) self-esteem (N=390). Note: Squares indicate observable questionnaire items; Ellipses indicate latent factors; Single-headed arrows indicate direct effects including measurement errors; Double-headed arrows indicate correlations; *P<0.05; **P<0.01.

Supplementary Table 3 Residual correlation matrices of the final measurement models for the mental functions anxiety, depression, optimism and self-esteem (N=390)

Measurement model and item	1.	2.	3.	4.	5.	6.	7.	8.	9.	10.	11.	12.	13.	14.	15.	16.	17.	18.	19.	20.	21.	22.	23.	24.
Anxiety																								
1. Stressed	0.00																							
2. Scared	-0.06	0.00																						
3. Worried	0.04	-0.01	0.00																					
4. Relaxed	0.03	-0.04	-0.05	0.00																				
5. Fearing	-0.05	0.05	-0.01	0.00	0.00																			
6. Restless	0.04	-0.01	-0.01	0.08	-0.08	0.00																		
7. Panicked	-0.01	0.00	0.00	0.00	0.01	-0.02	0.00																	
Depression																								
8. Enjoying as before								0.00																
9. Laughing								0.04	0.00															
10. Being cheerful								-0.03	0.03	0.00														
11. Slowed down								-0.02	-0.08	-0.01	0.00													
12. Interested in appearance								0.08	-0.03	-0.02	-0.04	0.00												
13. Looking forward								0.02	-0.02	-0.01	0.07	-0.02	0.00											
14. Enjoying a book								-0.10	0.02	0.04	0.05	0.00	-0.01	0.00										
Optimism																								
15. Expecting the best															0.00									
16. Things go wrong if they can															0.01	0.00								
17. Optimistic about future															0.00	-0.02	0.00							
18. Expecting things to go wrong															-0.04	0.01	0.04	0.00						
19. Not relying on good things															0.03	-0.01	-0.02	0.00	0.00					
20. Expecting good things															0.00	0.01	0.00	-0.02	0.01	0.00				
Self-esteem																								
21. Having good qualities																					0.00			
22. Feeling useless																					0.00	0.00		
23. Being of worth																					0.02	-0.05	0.00	
24. Taking a positive attitude																					-0.03	0.04	0.00	0.00

Supplementary Table 4 Measurement invariance tests of the measurement models for aetiology, age, sex, level and severity of injury and language (German, French) groups

Measurement model and level of invariance	Invariant?	χ^2_M	df_M	Model comparison	
				χ^2_D	df_D
		Aetiology			
Anxiety					
Configural	Y	26.389	28		
Weak	Y	36.130	34	6.478	6
Depression					
Configural	Y	26.486	26		
Weak	Y	37.071	32	6.664	6
Optimism					
Configural[a]	Y	6.185	10		
Weak[a]	N	25.577	15	20.180**	5
Self-esteem					
Configural	N[c]	-	-		
Weak	-	-	-	-	-
		Age			
Anxiety					
Configural	Y	23.869	28		
Weak	Y	31.491	34	5.139	6
Depression					
Configural	Y	23.256	26		
Weak	Y	27.580	32	3.339	6
Optimism					
Configural	Y	6.065	10		
Weak	Y	12.881	15	6.922	5
Self-esteem					
Configural	Y	5.893	2		
Weak	Y	10.112	5	5.158	3
		Sex			
Anxiety					
Configural	Y	22.071	28		
Weak	Y	33.311	34	6.856	6
Depression					
Configural[a]	Y	20.912	26		
Weak[a]	Y	31.261	32	6.616	6
Optimism					
Configural	Y	5.830	10		
Weak	N	21.025	15	12.035*	5
Self-esteem					
Configural	Y	3.280	2		
Weak	Y	9.258	5	7.138	3
		Language			
Anxiety					
Configural	Y	19.520	28		
Weak	Y	39.290	34	9.605	6
Depression					
Configural	Y	24.813	26		
Weak	Y	29.799	32	4.688	6
Optimism					
Configural	Y	9.786	10		
Weak	N	26.125	15	11.930*	5
Self-esteem					
Configural	Y	4.239	2		
Weak	Y	11.820	5	6.806	3

Abbreviations: M, Model; D, Difference; Y, Yes; N, No.
Note: Configural, no constrains on parameter estimates across groups; Weak, factor loadings constrained to be equal across groups; [a]Collapsing of two response categories needed for one factor indicator due to missing observations; [b]Collapsing of two response categories needed for two factor indicators due to missing observations; [c]Pattern of factor loading not equal across groups in terms of significance (P<0.05); *P<0.05; **P<0.01.

Supplementary Table 4 Continued

Measurement model and level of invariance	Invariant?	χ^2_M	df_M	Model comparison	
				χ^2_D	df_D
		Level of injury			
Anxiety					
Configural	Y	34.231	28		
Weak	N	55.579	34	12.598*	6
Depression					
Configural	Y	29.557	26		
Weak	Y	39.726	32	6.565	6
Optimism					
Configural	Y	6.059	10		
Weak	Y	9.063	15	3.511	5
Self-esteem					
Configural	Y	4.464	2		
Weak	Y	7.138	5	4.011	3
		Severity of injury			
Anxiety					
Configural[b]	N[c]	-	-		
Weak	-	-	-	-	-
Depression					
Configural	Y	30.602	26		
Weak	N	74.565	32	15.219*	6
Optimism					
Configural	N[c]	-	-		
Weak	-	-	-	-	-
Self-esteem					
Configural	N[c]	-	-		
Weak	-	-	-	-	-

Abbreviations: M, Model; D, Difference; Y, Yes; N, No.
Note: Configural, no constrains on parameter estimates across groups; Weak, factor loadings constrained to be equal across groups; [a]Collapsing of two response categories needed for one factor indicator due to missing observations; [b]Collapsing of two response categories needed for two factor indicators due to missing observations; [c]Pattern of factor loading not equal across groups in terms of significance (P<0.05); *P<0.05; **P<0.01.

Supplementary Table 5 Residual correlation matrices of the structural models (N=390)

Structural model and item	1.	2.	3.	4.	5.	6.	7.	8.	9.	10.	11.	12.	13.	14.	15.	16.	17.	18.	19.	20.	21.	22.	23.	24.	25.	26.	27.	28.
Anxiety																												
1. Stressed	0.00																											
2. Scared	-0.05	0.00																										
3. Worried	0.04	0.00	0.00																									
4. Relaxed	0.03	-0.04	-0.05	0.00																								
5. Fearing	-0.05	0.05	0.00	0.00	0.00																							
6. Restless	0.05	-0.01	-0.02	0.09	-0.07	0.00																						
7. Panicked	-0.01	0.00	0.01	-0.01	0.01	-0.01	0.00																					
8. Independence in performing activities of daily living	0.02	-0.02	-0.04	0.00	0.00	0.07	-0.02	0.00																				
Depression																												
9. Enjoying as before									0.00																			
10. Laughing									0.03	0.00																		
11. Being cheerful									-0.03	0.04	0.00																	
12. Slowed down									-0.02	-0.07	-0.02	0.00																
13. Interested in appearance									0.08	-0.06	-0.01	-0.04	0.00															
14. Looking forward									0.02	-0.03	-0.01	0.07	-0.02	0.00														
15. Enjoying a book									-0.10	0.03	0.03	0.05	0.00	0.00	0.00													
16. Independence in performing activities of daily living									-0.02	0.03	-0.01	-0.01	-0.02	0.00	0.03	0.00												
Optimism																												
17. Expecting the best																	0.00											
18. Things go wrong if they can																	0.00	0.00										
19. Optimistic about future																	0.00	0.00	0.00									
20. Expecting things to go wrong																	0.00	-0.03	0.01	0.00								
21. Not relying on good things																	-0.04	0.04	-0.01	0.01	0.00							
22. Expecting good things																	0.02	-0.02	0.00	0.01	0.00	0.00						
23. Independence in performing activities of daily living																	0.03	-0.01	-0.02	0.01	0.00	-0.01	0.00					
Self-esteem																												
24. Having good qualities																								0.00				
25. Feeling useless																								0.00	0.00			
26. Being of worth																								0.03	-0.06	0.00		
27. Taking a positive attitude																								-0.03	0.03	0.00	0.00	
28. Independence in performing activities of daily living																								-0.08	0.12	-0.03	0.00	0.00

Note: The structural models were set up conditional on the variables of the body structures, body functions and personal factors (exogenous covariates). Therefore, the model-implied residual correlation matrices include the variables of the respective mental functions and activities, only.
